# Social Media Communication and Network Correlates of HIV Infection and Transmission Risks Among Black Sexual Minority Men: Cross-sectional Digital Epidemiology Study

**DOI:** 10.2196/37982

**Published:** 2022-10-20

**Authors:** Lindsay E Young, Jack Lipei Tang, Yuanfeixue Nan

**Affiliations:** 1 Annenberg School for Communication and Journalism University of Southern California Los Angeles, CA United States

**Keywords:** HIV surveillance, HIV prevention, digital epidemiology, social media, social networks, sexual minority men, men who have sex with men

## Abstract

**Background:**

In the United States, HIV disproportionately affects Black cisgender sexual minority men (BSMM). Although epidemiological and behavioral surveillance are integral to identifying BSMM at risk of HIV infection and transmission, overreliance on self-reported data, inability to observe social contexts, and neglect of populations with limited engagement in health care systems limits their effectiveness. Digital epidemiological approaches drawing on social media data offer an opportunity to overcome these limitations by passively observing in organic settings activities, beliefs, behaviors, and moods that indicate health risks but are otherwise challenging to capture.

**Objective:**

The primary aim of this study was to determine whether features of Facebook communication and networks were associated with biological, behavioral, and psychological indicators of HIV infection and transmission risk.

**Methods:**

Facebook and survey data were collected from BSMM aged 18 to 35 years living in Chicago (N=310). Participants’ Facebook posts were characterized using 4 culturally tailored topic dictionaries related to aspects of HIV protection and risk among BSMM (sexual health; substance use; sex behavior; and ballroom culture, a salient subculture in lesbian, gay, bisexual, transgender, and queer communities of color). Social network methods were used to capture structural features of BSMM’s Facebook friendships (centrality, brokerage, and local clustering) and Facebook group affiliations. Multivariable regressions revealed relationships between these Facebook features and 5 ground truth indicators of HIV infection and transmission risk (sexually transmitted infection incidence, condomless sex, sex drug use, biomedical prevention, and depression).

**Results:**

Although analysis of participants’ Facebook posts revealed that HIV-related topics occupied a small portion of the total messages posted by each participant, significant associations were found between the following HIV risk indicators and Facebook features: *Condomless sex*, including communication about sexual health (odds ratio [OR] 1.58, 95% CI 1.09-2.29), ballroom culture (OR 0.76, 95% CI 0.63-0.93), and friendship centrality (OR 0.69, 95% CI 0.52-0.92); *Sex drug use*, including communication about substance use (OR 1.81, 95% CI 1.17-2.79) and friendship centrality (OR 0.73, 95% CI 0.55-0.96) and brokerage (OR 0.71, 95% CI 0.51-0.99); *Biomedical prevention*, including communication about ballroom culture (OR 0.06, 95% CI 0.01-0.71); and *Depression*, including communication about sexual health (*β*=–0.72, 95% CI −1.42 to −0.02), ballroom culture (*β*=.80, 95% CI 0.27-1.34), friendship centrality (*β*=−0.90, 95% CI −1.60 to −0.21), and Facebook group affiliations (*β*=.84, 95% CI 0.25-1.43). Facebook features provided no significant explanatory value for sexually transmitted infection incidence.

**Conclusions:**

Finding innovative strategies to detect BSMM at risk of contracting or transmitting HIV is critical to eliminating HIV disparities in this community. The findings suggest that social media data enable passive observance of social and communicative contexts that would otherwise go undetected using traditional HIV surveillance methods. As such, social media data are promising complements to more traditional data sources.

## Introduction

### Background

Despite significant strides in preventing and treating HIV in the United States, the burden of HIV remains disproportionately concentrated among cisgender gay, bisexual, same gender–loving, and other sexual minority men (SMM), of which Black or African American SMM (BSMM) demonstrate particular vulnerability. Of the estimated 34,800 new HIV infections in 2019, a total of 66% (23,100) were among SMM, with BSMM accounting for the plurality (37%) of those cases [1] despite comprising only approximately 9% of the SMM population [2]. With greater prevalence of HIV among BSMM comes greater opportunity for its transmission within BSMM networks as BSMM tend to select sexual partners of the same race [3]. Opportunities for transmission are further compounded by the fact that many BSMM are unaware of their HIV infection [4], in part because of underestimations of personal risk and limited access to high-quality HIV prevention services [5]. People who do not know that they have HIV do not receive timely medical care and unknowingly infect others.

At the patient level, the risks of both HIV infection and transmission are bound in a web of intersecting and mutually exacerbating biological, behavioral, and psychosocial factors. Biologically, HIV risk cannot be discussed in isolation from other sexually transmitted infections (STIs), which are known to increase susceptibility to and transmission of HIV given their shared pathways of transmission [6]. Behaviorally, engagement in unprotected anal intercourse (hereafter, condomless sex) and other sex behaviors that can compromise condom use decisions, for example, use of drugs during sex (hereafter, sex drug use), account for most new infections and transmission instances [7]. Alternatively, protective biomedical behaviors such as adherence to daily pre-exposure prophylaxis (PrEP) for HIV-negative individuals (ie, PrEP as prevention) and antiretroviral medications for people living with HIV (ie, treatment as prevention) are viewed as highly efficacious HIV prevention practices [8]. However, linkage and retention in both regimens of HIV prevention are lower among BSMM relative to their White counterparts, in large part because of systemic vulnerabilities (eg, poverty, housing instability, medical mistrust, and structural racism) that create barriers to medication adherence [9-12].

Finally, adverse mental health conditions such as depression and low self-esteem are also correlates of HIV risk through their associations with high-risk behaviors such as condomless sex, substance use, and multiple concurrent partnerships [13-15]. Depression may increase the likelihood of engaging in these behaviors to mitigate negative feelings brought on by HIV stigma, homophobia, and discrimination [16] and may compromise motivations to change these behaviors [14]. Moreover, depression prevalence among people living with HIV can compromise their ability to remain adherent to HIV medications and, therefore, achieve sustained viral suppression [17,18].

### Traditional HIV Risk Surveillance and Its Limitations

Key to efforts to attenuate the HIV epidemic among SMM of all races and ethnicities is the ability to actually observe the aforementioned indicators of HIV risk in individuals before moments of infection and transmission such that prevention can actually occur [19,20]. Traditionally, researchers and public health professionals have relied on surveillance data that suffer from considerable lag time in reporting as they are often captured through large-scale behavioral surveys or clinical case visits, which require extensive resources for collection and analysis [21,22]. Perhaps more importantly, with their reliance on surveys and clinical visits, traditional surveillance strategies inevitably neglect populations that have limited engagement with the health care system because of issues such as low perceptions of personal risk, experiences with stigma and discrimination, and socioeconomic disadvantage. With studies suggesting limited access to health care services for BSMM, including HIV care [23], data predicated on an individual’s use of health care services may not represent those at greatest risk of contracting or unknowingly transmitting HIV or those who are most vulnerable to falling out of care once diagnosed with HIV. Finally, with their emphasis on individual risk behaviors, survey- and interview-based surveillance methods are not well suited to observe the social contexts that nurture behavioral risk, such as social networks [24], social and behavioral norms [25], and peer-group dynamics [26].

### Using Social Media to Surveil Social and Communicative Contexts Associated With HIV Risks

More recently, the mainstreaming of social media and other digital communication technologies has given way to the emerging field of *digital epidemiology* [22,27]. Digital epidemiology rests on the idea that digital communications such as social media posts are made in organic environments and that what people post about reflects activities, behaviors, and dispositions that are relevant to various health outcomes. Demonstration studies have put these assumptions to the test and showed that what people talk about on social media platforms can be used to predict health outcomes such as depression [28], sexual risk [29], and HIV and STI *hotspots* [30].

Moreover, digital epidemiology is poised to take advantage of the networked (or relational) infrastructure that undergirds social media to detect features of interpersonal networks through which HIV risks are conferred [31,32]. For example, previous research focusing on offline networks has shown that the cohesion of an individual’s network [33,34], an individual’s position vis-à-vis other network members [24], and their exposure to other risky individuals [35-38] affect disease spread and risk susceptibility. In addition, looser affiliations formed around broader groups or organizational structures have also been shown to nurture and confer HIV risks [31], for example, physical-world social venues (eg, bars, clubs, and bathhouses) [39-44] and their digital analogs (eg, dating apps and lesbian, gay, bisexual, transgender, and queer social networking groups) [45,46].

### Objectives

Given the need to engage more high-risk BSMM in the HIV prevention and treatment continuum of care and known limitations of traditional approaches to HIV risk surveillance, it is essential to understand whether alternative, more organic sources of social and behavioral information can be used to improve surveillance of HIV risk among BSMM. To this end, we took an initial step to evaluate the feasibility of using digital traces of BSMM’s Facebook communication and network connections as an additional source of informal health information typically not reported to medical officials or health departments [47]. Specifically, we sought to ascertain (1) the extent to which BSMM posted on Facebook about topics known to be associated with HIV prevention and risk in their community and (2) the extent to which those topics and the features of their Facebook friendship connections were associated with biological, behavioral, and psychological indicators of HIV infection and transmission risk.

## Methods

### Participants

This study draws on parent study data collected from 2016 to 2018 from participants enrolled in a social network PrEP for prevention intervention for BSMM living in Chicago, Illinois, United States. As described elsewhere [48,49], participants were eligible to take part in the parent study if they (1) were aged 18 to 35 years; (2) identified as Black or African American; (3) were assigned male at birth; (4) had had sex with a man in the past 12 months; and, because of our interest in social media use, (5) had an active Facebook profile. Participants were recruited using a probability-based variant of snowball sampling called respondent-driven sampling (RDS) [50]. RDS referral chains began with an initial set of “seeds” meeting study eligibility who were then enlisted to recruit up to 6 of their peers (“sprouts”) who also met study eligibility. The process continued until the recruitment target was reached (N=423).

The analyses featured in this study draw on data collected at the parent study’s 12-month assessment as this was the only assessment that included questions about sexual risk behavior. In total, 82% (347/423) of the parent study participants were retained at 12 months, and only those who had complete data on all variables of interest (310/347, 89.3%) were included in our analyses. Bivariate analyses (not shown) comparing participants included in the analytic sample (310/347, 89.3%) with filtered cases (37/347, 10.7%) showed no significant differences in age, education, sexual identity, or HIV status.

### Data Sources and Measures

#### Overview

All study measures were derived from 3 sources of data obtained with consent from participants: (1) a computer-assisted self-administered survey, which included modules on HIV prevention engagement, sex behaviors, substance use, and demographics; (2) biomedical testing, which determined participants’ HIV status and viral load if they were HIV positive; and (3) a manual download of participants’ Facebook friend lists, group memberships, and timeline data, which enabled the construction of their web-based friendship and group affiliation networks and analysis of their public communication content, respectively.

#### Outcomes

The outcomes included 5 indicators of HIV infection and transmission risk that spanned biological, behavioral, and psychosocial domains. As a biological indicator of risk, we included a measure of recent *STI incidence,* which was operationalized dichotomously as whether a participant self-reported having been diagnosed with at least one STI other than HIV (eg, syphilis, chlamydia, or human papillomavirus) in the past 12 months. Behavioral indicators included measures of condomless sex, sex drug use, and biomedical prevention engagement. Engagement in *condomless sex* (ie, sex without a condom) and *sex drug use* (ie, using drugs or alcohol to enhance the sexual experience or make sex easier) were defined dichotomously as inconsistent condom use (ie, not always using a condom) and having ever engaged in sex drug use in the past 6 months. *Biomedical prevention engagement* was operationalized as either being on PrEP to prevent contracting HIV or achieving viral suppression to prevent transmitting HIV [6]. As such, the measure of biomedical prevention engagement is intentionally status neutral, and not being engaged in this form of prevention is taken to be the indication of risk. *Depression* was included as a psychosocial indicator of risk and was measured using the Revised Center for Epidemiologic Studies Depression Scale [51], which consists of 10 items (Cronbach α=.81), each investigating the frequency of a depressive feeling within the last week (0=“rarely or none of the time” to 3=“all of the time”). Item scores were aggregated to create a composite depression score.

#### Facebook Communication Features

To capture the degree to which participants posted about topics that have been linked to HIV protections and vulnerabilities, we constructed a series of topical dictionaries to enable an automated content analysis of their timeline posts. To develop each dictionary, an iterative, mixed methods approach was used, described in greater detail in Multimedia Appendix 1 [52-54], that drew on extant literature, the expertise of BSMM themselves, internet sources, and endogenous patterns in the data themselves. Topics featured in this study include *sexual health*; *substance use*; *sex behavior*; and *house/ballroom culture*, a subaltern system of chosen family (also known as *Houses*) and identity-affirming competitions (also known as *Balls*) that feature prominently in the Black gay community and that are thought to provide sources of support that help buffer BSMM members from HIV vulnerabilities such as financial hardship, housing instability, and stigma and discrimination [52-54]. Example terms and posts associated with each topic dictionary are shown in Table 1. Using the flagged posts, we then created 4 participant-level topic variables representing the number of posts a participant made with a given topic orientation. In addition, we accounted for the *positive affec*t of posts, which has been linked to HIV-adjacent outcomes such as depression [28] and substance use [55]. Positive affect was measured using the Linguistic Inquiry and Word Count (Pennebaker Conglomerates, Inc) program [56].

**Table 1:** Topic dictionaries.

Topics	Example keywords	Sample post	Posts, n (%)
Sexual health	*HIV, AIDS, condom, PrEP, PEP, sexually transmitted, STD, get tested,* and *poz*	“condoms have become my best friend kinda sorta”	420 (0.18)
Sex behavior	*Sex, bareback, masturbate, blow job, give head, cock, horny,* and *thirst trap*	“I’m bored horny with nothing and nobody to do lmaooooooo this isn’t happening to me right now lol”	3452 (1.5)
Substance use	*Drugs, meth, poppers, weed, marijuana, liquor, alcohol, boozie, turnt,* and *getting loud*	“i went out last night turnt up fell asleep woke up still turnt”	3754 (1.63)
Ballroom culture	House names: *Balenciaga, Mizrahi,* and *Cartier;* status and roles: *legends, icons,* and *house mother*; competition categories: *realness, sex siren,* and *vogue*	“rules of twist you must walk your realness category in order to get respected as a twister if you don’t walk realness please don’t think im gone let you sit next to me in the twist line you cn vouge wit the vouge fems”	4510 (1.96)

#### Facebook Network Features

In line with previous work showing associations between how SMM are positioned vis-à-vis one another in their social networks and their prevention and risk engagement [24,57], we also accounted for 4 measures of an individual’s web-based structural embeddedness in a Facebook friendship network among the other BSMM in the study. *Eigenvector centrality* measures the degree to which an individual is connected with other well-connected network members and, hence, their relative closeness to others in the network [58,59]. *Network brokerage* represents the degree to which an individual connects disparate subcommunities within the friendship network and was measured using the brokerage measure by Everett and Valente [60]. A measure of *local clustering* [61] was also included to represent the degree to which actors in a network create social cliques. Finally, *group network size* accounts for an individual’s group-mediated embeddedness and was operationalized as the number of Facebook groups to which an individual belonged.

#### Controls

The models were adjusted for several control measures, including (1) a dichotomous measure of HIV status (1=HIV positive; 0=HIV negative) defined based on blood tests for those who consented to blood tests or self-reports for those who opted out, (2) a measure of the total number of posts made in the past 12 months to control for the varying volume of posts across participants, and (3) the other 4 indicators of HIV infection and transmission risk.

### Statistical Analysis

Descriptive statistics and multivariable logistic (for dichotomous outcomes) and linear (for numeric outcomes) regression models were estimated using RStudio [62] (version 1.4.1717; R Foundation for Statistical Computing). Each outcome was regressed on the same set of factors, including the Facebook communication and network features and the other HIV prevention and risk outcomes. In addition, all numeric (interval or ratio) covariates were standardized for ease of interpretation. The effects in the logistic regression models are reported as odds ratios (ORs), whereas the coefficients are reported in the linear regression model of depression. RDS sampling weights were not included in the regressions as this would amount to assuming heteroskedasticity, where respondents with high weights would be assumed to provide the most accurate information. There is no reason for such an assumption.

### Ethical Considerations

All study procedures were approved by the Institutional Review Board of the University of Chicago (IRB15-1250). Informed consent was obtained from all the participants. For all nonparticipant Facebook friends of study participants, a waiver of consent was obtained from the Institutional Review Board given the minimal risk to these individuals. The parent randomized trial is registered at ClinicalTrials.gov (identifier NCT02896699).

## Results

### Descriptives

Participants in the analytic sample were, on average, aged 25.8 (SD 4.21) years. Most had earned a high school–level education (193/310, 62.3%) and identified as gay (188/310, 60.6%), whereas a quarter identified as bisexual (80/310, 25.8%). Summary statistics of key variables in our analysis are provided in Table 2. Of the 310 BSMM in the analytic sample, 147 (47.4%) were living with HIV, 69 (22.3%) reported an STI diagnosis in the past 12 months, 103 (33.2%) were either on PrEP or were virally suppressed (ie, biomedical prevention engagement), 193 (62.3%) reported condomless sex, and 149 (48.1%) reported sex drug use. A Revised Center for Epidemiologic Studies Depression Scale score of 10 is considered depressed; on average, participants were slightly below that threshold (mean 9.30, SD 5.94; range 0-28).

Analysis of participants’ Facebook posts revealed that HIV-related topics accounted for a small portion of the total messages posted by participants (mean 730.63, SD 836.79). Overall, participants posted a median of 4 messages (IQR 1-14) about substance use, a median of 3 messages (IQR 0-12) about sex behavior, a median of 2 messages (IQR 0-11) about ballroom culture, and a median of 0 messages (IQR 0-2) about sexual health (Table 2).

**Table 2:** Summary statistics of HIV infection and transmission risk indicators and Facebook communication and network features of Black sexual minority men (N=310).

Characteristic			Values
**HIV infection and transmission risk indicators**			
	STI^a^ incidence, n (%)	69 (22.3)	
	Condomless sex, n (%)	193 (62.3)	
	Sex drug use, n (%)	149 (48.1)	
	Biomedical prevention engagement, n (%)	103 (33.2)	
	Depression score, mean (SD)	9.30 (5.94)	
**Facebook communication features**			
	Sexual health posts, median (IQR)	0 (0-2)	
	Substance use posts, median (IQR)	4 (1-14)	
	Sex behavior posts, median (IQR)	3 (0-12)	
	Ballroom culture posts, median (IQR)	2 (0-11)	
	Positive affect, mean (SD)	4.84 (2.42)	
**Facebook network features**			
	Eigenvector centrality, mean (SD)	0.17 (0.16)	
	Brokerage, mean (SD)	28.67 (36.41)	
	Local clustering, mean (SD)	0.16 (0.10)	
	Facebook group count, mean (SD)	58.24 (64.98)	
	HIV-positive status, n (%)	147 (47.4)	
	Total number of posts, mean (SD)	730.63 (836.79)	

^a^STI: sexually transmitted infection.

### Associations Between Facebook Features and HIV Infection and Transmission Risks

The multivariable regressions of the HIV infection and transmission risk indicators are shown in Table 3. First, the logistic regression of STI incidence revealed that, once the positive and significant effects of engagement in biomedical prevention and condomless sex were accounted for, Facebook network and communication features offered no additional explanatory power.

Regarding condomless sex, individuals who posted about sexual health had greater odds of reporting condomless sex in the past 6 months (OR 1.58, 95% CI 1.09-2.29), whereas posting about aspects of ballroom culture decreased those odds (OR 0.76, 95% CI 0.63-0.93). Facebook network features were also revealing. Individuals who were connected with other well-connected BSMM (ie, eigenvector centrality) had decreased odds of engaging in condomless sex (OR 0.69, 95% CI 0.52-0.92). A negative trend was also observed for network brokerage (ie, connecting disparate subcommunities of BSMM on Facebook), although the relationship was not statistically significant (*P*=.07). Among the other HIV risk indicators, only STI incidence was positively associated with condomless sex (Table 3).

The model of sex drug use revealed several pronounced effects of Facebook network and communication features. Among the communication topics, individuals who posted about substances were more likely to report using drugs during sex (OR 1.81, 95% CI 1.17-2.79), whereas adopting a more positive affective tone in posts decreased those odds (OR 0.73, 95% CI 0.55-0.96). With respect to network features, individuals who were Facebook friends with other well-connected BSMM (ie, eigenvector centrality; OR 0.73, 95% CI 0.55-0.96) and those who bridged disparate subcommunities of BSMM on Facebook (ie, brokerage; OR 0.71, 95% CI 0.51-0.99) were less likely to report sex drug use. Among the other HIV risk indicators, depression was positively associated with sex drug use (Table 3).

With respect to biomedical prevention engagement, after controlling for the positive and significant effects of HIV status and STI incidence, the results showed that individuals who posted more often about aspects of ballroom culture were less likely to be on PrEP or achieve viral suppression through antiretroviral therapy adherence (OR 0.06, 95% CI 0.01-0.71). Meanwhile, individuals who posted about sexual health were more likely to adhere to biomedical prevention (*P*=.05), although this trend did not meet the criterion of significance.

Finally, regarding depression, individuals who posted about sexual health tended to be less depressed (*β*=−0.72, 95% CI −1.42 to −0.02), whereas those who posted about ballroom culture tended to be more depressed (*β*=.74, 95% CI 0.11-0.37). Furthermore, posting about substance use (*P*=.07) and sex behavior (*P*=.06) showed negative and positive trends, respectively, albeit nonsignificant ones. Of the Facebook network features, being connected with other well-connected BSMM on Facebook (ie, eigenvector centrality; *β*=−0.90, 95% CI −1.60 to −0.21) and being affiliated with more Facebook groups (*β*=.84, 95% CI 0.25-1.43) were negatively and positively associated with depression, respectively. Sex drug use was also positively associated with depression (Table 3).

**Table 3:** Multivariable logistic and linear regression models to assess associations between Facebook communication and network features and HIV infection and transmission risk indicators (N=310).

Independent variables		STI^a^ incidence^b^, OR^c^ (95% CI)	Condomless sex^b^, OR (95% CI)	Sex drug use^b^, OR (95% CI)	Biomedical prevention^b^, OR (95% CI)	Depression^d^, β (95% CI)
**Facebook communication features**						
	Sexual health content (std^e^)	0.99 (0.73 to 1.33)	1.58 (1.09 to 2.29)^f^	1.22 (0.89 to 1.67)	1.48 (1.00 to 2.20)^g^	−0.72 (−1.42 to −0.02)^f^
	Substance use content (std)	1.06 (0.72 to 1.56)	1.13 (0.81 to 1.58)	1.81 (1.17 to 2.79)^h^	0.89 (0.58 to 1.36)	−0.80 (−1.65 to 0.05)^g^
	Sex behavior content (std)	0.88 (0.66 to 1.18)	1.02 (0.72 to 1.43)	1.02 (0.73 to 1.43)	1.20 (0.82 to 1.77)	.63 (−0.03 to 1.04)^g^
	Ballroom culture content (std)	1.24 (0.92 to 1.66)	0.76 (0.63 to 0.93)^h^	0.94 (0.76 to 1.18)	0.06 (0.01 to 0.71)^f^	.80 (0.27 to 1.34)^h^
	Positive affect (std)	0.81 (0.56 to 1.15)	1.08 (0.83 to 1.42)	0.73 (0.55 to 0.96)^f^	0.85 (0.66 to 1.08)	.18 (−0.45 to 0.81)
**Facebook network features**						
	Eigenvector centrality (std)	1.22 (0.88 to 1.70)	0.69 (0.52 to 0.92)^f^	0.73 (0.55 to 0.96)^f^	0.97 (0.69 to 1.36)	−0.90 (−1.60 to −0.21)^f^
	Brokerage (std)	0.97 (0.61 to 1.54)	0.74 (0.53 to 1.03)^g^	0.71 (0.51 to 0.99)^f^	0.78 (0.44 to 1.37)	−0.44 (−1.29 to 0.42)
	Local clustering coefficient (std)	0.96 (0.61 to 1.51)	0.78 (0.53 to 1.15)	0.89 (0.60 to 1.30)	1.13 (0.76 to 1.66)	.01 (−1.03 to 1.04)
	Facebook group count (std)	0.82 (0.60 to 1.13)	1.00 (0.78 to 1.30)	1.01 (0.79 to 1.29)	0.85 (0.66 to 1.10)	.84 (0.25 to 1.43)^h^
**Controls**						
	HIV status (positive)	1.84 (0.97 to 3.47)^g^	0.90 (0.53 to 1.52)	0.85 (0.51 to 1.40)	3.20 (1.85 to 5.53)^i^	.30 (−1.02 to 1.62)
	Total number of posts (std)	1.03 (0.62 to 1.72)	0.75 (0.50 to 1.11)	0.64 (0.40 to 1.03)^g^	1.12 (0.68 to 1.84)	.65 (−0.60 to 1.90)
	STI incidence	—^j^	2.66 (1.40 to 5.04)^h^	1.00 (0.54 to 1.87)	2.65 (1.43 to 4.89)^h^	.27 (−1.34 to 1.87)
	Condomless sex	2.71 (1.44 to 5.55)^h^	—	1.19 (0.70 to 2.01)	0.90 (0.52 to 1.57)	−0.12 (−1.57 to 1.32)
	Sex drug use	1.00 (0.51 to 1.72)	1.18 (0.70 to 2.00)	—	1.02 (0.59 to 1.76)	2.69 (1.27 to 4.11)^i^
	Biomedical prevention	2.59 (1.42 to 4.73)^h^	0.85 (0.49 to 1.48)	0.92 (0.53 to 1.58)	—	−0.34 (−1.70 to 1.02)
	Depression (std)	1.05 (0.77 to 1.44)	0.98 (0.76 to 1.27)	1.64 (1.27 to 2.11)^i^	0.90 (0.69 to 1.17)	—
	Intercept	0.07 (0.03 to 0.15)^i^	1.46 (0.91 to 2.33)	0.96 (0.59 to 1.57)	0.17 (0.09 to 0.35)^i^	8.05 (6.58 to 9.52)^i^

^a^STI: sexually transmitted infection.

^b^Logistic regression model.

^c^OR: odds ratio.

^d^Linear regression model.

^e^Std: SD unit change.

^f^*P*<.05.

^g^*P*<.10.

^h^*P*<.01.

^i^*P*<.001.

^j^Outcome variable; excluded as a covariate.

### HIV Status Subgroup Analysis of Engagement in Biomedical Prevention

In the primary analysis, we prioritized a status-neutral measure of biomedical prevention engagement in light of evidence that, similar to PrEP, viral suppression through antiretroviral therapy adherence is itself an effective form of biomedical HIV prevention (ie, treatment as prevention) [63,64]. Despite this and calls to prioritize a status-neutral continuum of care, we acknowledge that the circumstances that enable medication initiation and adherence may be different for people living with HIV than for people at risk of HIV.

For this reason, we performed a stratified subgroup analysis to determine whether Facebook features were differently associated with each subgroup’s likelihood of being engaged in biomedical forms of HIV health care (Multimedia Appendix 2). As shown, the negative association between posting about ballroom culture and biomedical engagement that was observed in the primary analysis was only observed among BSMM living with HIV in the stratified analysis. As such, BSMM living with HIV who showed signs of identifying with the ballroom community were less likely to be virally suppressed. In addition, the positive association between STI incidence and biomedical engagement that we observed in the unstratified model was shown to be significant only among HIV-negative BSMM.

### Improvements in Model Fit

We performed likelihood ratio tests for all binary outcomes and hierarchical regression for the continuous outcome of depression to determine whether the addition of Facebook features to models with the HIV risk indicators only improved model fit (Table 4). Variables that met a *P*<.10 Cronbach α criterion in the primary analysis (Table 3) were included in this portion of the analysis. For each outcome, eligible HIV risk indicators were included in model 1, with eligible Facebook features added to that in model 2. As shown, with the exception of STI incidence (not shown), for which Facebook features provided no additional explanatory power in the primary analysis, the addition of Facebook features significantly improved model fit over models with HIV risk indicators alone. For condomless sex, biomedical prevention engagement, and depression, model improvements were significant at the *P*<.01 level. For sex drug use, model improvement was significant at the *P*<.001 level.

**Table 4:** A comparison of model fits: models with indicators of HIV infection and transmission only (model 1) versus models with Facebook features added (model 2).

Dependent variable and model		LR^a^ chi-square (*df*)	*F* test (*df*)	Model 1 vs Model 2 LR chi-square (*df*)	Model 1 vs Model 2 *F* change (*df*)	*P* value
**Condomless sex**			N/A^c^	18.2 (4)^d^		.001
	Model 1^b^	6.8 (1)				
	Model 2^e^	25.0 (5)				
**Sex drug use**			N/A	21.4 (5)^d^		<.001
	Model 1	16.5 (1)				
	Model 2	37.9 (6)				
**Biomedical prevention**			N/A	11.5 (2)^d^		.003
	Model 1	31.6 (2)				
	Model 2	43.2 (4)				
**Depression^f^**		N/A		N/A	3.14 (6, 302)^d^	.005
	Model 1		16.88 (1, 308)			
	Model 2		5.21 (7, 302)			

^a^LR: likelihood ratio.

^b^For each outcome, model 1 includes all HIV infection and transmission risk indicators that met a Cronbach α=.10 criterion in previous models.

^c^N/A: not applicable.

^d^The statistical significance of the comparison of the two models.

^e^For each outcome, model 2 adds to model 1 the Facebook network and communication variables that met a Cronbach α=.10 criterion in previous models.

^f^Depression is a numeric measure, so an *F* test and *F* change are reported instead of LR chi-square tests.

## Discussion

### Principal Findings

In this study, we argue that digital epidemiology—the practice of drawing on digital traces of web-based communication and social interaction to detect individuals with certain health risks—offers a critical step forward in closing the gap on HIV disparities as it may yield informal health information not otherwise found in more formal medical records and offers a way to detect at-risk individuals whose medical records are sparse in the first place. To test these assumptions, we demonstrated the potential of using Facebook communication and network data collected from a cohort of young BSMM, a population experiencing a disproportionate burden of new HIV incidences in the United States, to identify individuals who reveal key biological, behavioral, and psychological indicators of HIV infection and transmission risk. The fact that we were able to detect significant relationships between Facebook communication and network features and multiple HIV risk indicators while also adjusting for correlates of those outcomes that were directly observed using surveys and blood tests suggests that the effects of these social media variables are relatively robust.

Our demonstration yielded several insights about contingencies of the digital epidemiology approach in general and, more specifically, the relationship between various social media features and HIV risk indicators in this particular cohort of BSMM. Regarding the contingencies of the approach, although we intentionally examined 5 risk indicators that represented different aspects of HIV risk (ie, biological, behavioral, and psychosocial), our results clearly showed that Facebook communication and network features were not universally predictive of these outcomes. For example, features of network embeddedness, specifically attenuated social integration with other BSMM, were strong indicators of sex behaviors that can place BSMM at risk of HIV (ie, condomless sex and sex drug use) and mental health risks such as depression. The same was not true for biological risks such as STI incidence or for engagement in biomedical forms of HIV care, for which the more directly observed epidemiological and behavioral variables were far better predictors.

Regarding our statistical findings, several relationships stand out that can bring additional focus to these contingencies. A first set of noteworthy findings pertains to the effects of being embedded in web-based friendships with other BSMM. We learned that web-based friendships with other BSMM, both in the form of being connected with well-connected others and being a network bridge, were associated with decreased likelihoods of engaging in sex drug use and condomless sex. This suggests that those who engage in these sex behaviors may be at risk of becoming disenfranchised from the larger BSMM community, a finding we have found support for elsewhere in work currently under review. This raises concern as the isolation of already at-risk behavioral subgroups may lead to the adoption of additional compounding risk behaviors [65,66]. Therefore, public health outreach to these behavioral communities may need to incorporate ways to integrate them into the larger social fabric of BSMM, which will increase their access to social support from other members of the BSMM community and expose them to alternative behavioral norms and a more diverse range of behavioral choices. Social isolation among BSMM who engage in sex behaviors that place them at risk of HIV infection and transmission is also concerning given its obvious implications for facilitating or exacerbating depression [67], which itself had an unambiguous direct effect on the likelihood of using sex drugs.

The impact of social embeddedness also surfaced in the model of depression. Confirming our previous assertions about the relationship between social isolation (vis-à-vis other BSMM) and depression, we found that the BSMM in our sample who had fewer Facebook friendships with other well-connected BSMM (ie, had lower eigenvector centrality) tended to score higher on depression. Furthermore, and somewhat surprisingly, we also found that BSMM who belonged to more Facebook groups also tended to be more depressed. We surmise that this relationship is either the result of compensation behavior, whereby BSMM who are more depressed tend to seek social connection through group settings to help them reduce their depression—perhaps to compensate for their attenuated integration with other BSMM via friendships—or the result of negative interactions that occur in Facebook groups that may exacerbate negative mindsets. That our analysis was only cross-sectional and not longitudinal means that the directionality of this and all other relationships discussed cannot be adequately ascertained.

Second, there were significant relationships between posting about substance use and using drugs to enhance the sexual experience (ie, sex drug use) and a trending association between posting about sexual health and being engaged in sexual health care (ie, biomedical prevention). This supports the general intuition that most people have about communication on general-purpose platforms such as Facebook—that there tends to be a strong correlation between the behaviors that one talks about in digital spaces and the behaviors that one engages in real-life spaces. In many ways, the naturalistic settings in which posts on social media platforms are made (eg, at home, in school, and socializing with friends) mean that what one communicates is likely to reflect behaviors that are relevant to their routine activities and thinking [28]. That said, observing HIV risk behaviors and activities can be resource-intensive and can suffer from serious lags relative to when the moment of risk occurred when retrospective self-reports are used. Thus, knowing that digitally archived social media communication could be leveraged to detect at-risk individuals in place of behavioral observations or self-reports is an exciting public health advancement that could make detecting individuals at risk of HIV incidence or transmission and other comorbidities easier and more responsive to real-time moments of risk.

Finally, our results also suggest that communication on social media can be used to detect BSMM subcommunities such as the ballroom community, which tends to attract individuals who are already structurally vulnerable because of limited access to medical care, socioeconomic disadvantages, housing instability, experiences with rejection from biological families, and stigma and discrimination [54]. These factors may then induce additional HIV risk behaviors such as sex drug use [68] and survival sex [52], which can further increase their HIV risk. Our analysis confirmed this in 2 ways. Posting about aspects of ballroom culture was negatively associated with the likelihood of being on PrEP if HIV negative or virally suppressed if living with HIV and positively associated with heightened depression. Thus, although the ballroom community undoubtedly provides its members with kinship and a sense of sexual and gender affirmation, those drawn to it may be more likely to demonstrate pre-existing hardships and vulnerabilities that continue to affect the degree of HIV risk and prevention that they engage in or are exposed to within the ballroom community. As such, social media communication does more than reflect behaviors such as substance use. Its ability to detect members of sociocultural milieus with other known HIV vulnerabilities is also a critical advancement in HIV risk-reduction strategies.

### Clinical Implications

Our results suggest that social media data could be leveraged to improve surveillance and modeling of certain types of HIV infection and transmission risk among BSMM while also bringing our attention to new digital “fingerprints” that could serve as early warning signs of an individual’s HIV risk potential. Although the utility of digital epidemiological approaches is often discussed in the context of developing automated public health tracking systems at scale with large amounts of publicly available social media data, we argue for a more person-focused application of social media–assisted surveillance that can lead to more personalized forms of intervention and care. For instance, with knowledge of important digital fingerprints of potential risk, social media archives from consenting clients could help frontline health and social welfare staff (eg, HIV counselors, community health workers, and case managers) profile their clients in terms of their risk of future HIV-related outcomes, thereby serving as a barometer to guide their decisions on screening, treatment, and other service recommendations. Furthermore, having the ability to monitor extreme changes in a client’s communication and relational dynamics (eg, changes in mood or suddenly dissolved friendships), which have been linked in previous work to sexual risk engagement [69], can alert frontline staff to potential crises that warrant impromptu “soft touch” interventions in the form of supportive check-ins. As such, we see social media’s epidemiological promise in its ability to enable more responsive care and the ability to intervene in risky social contexts as they unfold in near real time.

### Limitations

This study represents an important first step in determining the efficacy of using digital epidemiological approaches to identify individuals at risk of HIV incidence or transmission. However, it is indeed just that, a first step. As such, its current limitations correspond to obvious next steps. Foremost of those limitations is that the data we used in this study are cross-sectional, which limits us to making correlational as opposed to causational inferences. A next step in our research agenda is to build predictive models that draw on all 3 waves of data from all 3 sources (ie, Facebook, surveys, and laboratory tests) using logistic and machine learning approaches.

Second, although the featured analyses account for the effects of both self-reported and passively observed social media indicators of HIV infection and transmission risk potential, it remains to be seen whether social media indicators on their own would be more helpful than self-reported health and behavioral data. Our intuition suggests that this would not be the case. Our results showed that passively observed social media indicators of an individual’s risk potential improved the predictive performance of models that included self-reported data alone, but we remain dubious about the prospects of using social media data as replacements for self-reported data. Rather, they are more appropriately seen as complements of one another.

Third, dictionary-based approaches to digital content analysis are limiting in that they only identify topically relevant keywords (as opposed to deeper meanings) and necessarily rely on the researchers’ ability to build a robust dictionary. Furthermore, as themes are defined by the researcher before performing the content analysis, more emergent themes and topics that may be related to HIV risk go unidentified and unexplored. Latent space topic modeling such as latent Dirichlet allocation would be an appropriate modeling approach to those ends. However, we are cautious about expressing too much enthusiasm for this approach, particularly when applied to Facebook content. Unlike Twitter, which often engenders a more intentional and focused tone, Facebook posts tend to be more diffuse and stream of consciousness, which we surmise will make detecting meaningful latent topics more challenging in these models.

### Conclusions

To end the HIV epidemic in the United States, ambitious goals have been set to reduce the number of incident infections by 90% by 2030, with prioritized intervention among BSMM [8]. Meeting this goal demands an innovative and multipronged strategy to identify individuals who are at high risk of HIV infection and transmission and engage them in prevention or treatment care continuums. Although well-funded epidemiological and behavioral surveillance programs are and should remain the primary engine of this work, they do not come without limitations. This study established that social media offers a complementary informal source of health information that can be used to sharpen our ability to detect individuals at risk of HIV and reach people who may otherwise be missed by surveillance that privileges those who engage more regularly with the mainstream health care system. Indeed, our analysis showed that social media communication and network features are correlates of several indicators of HIV infection and transmission risk among BSMM in our sample, although not uniformly. Moreover, the inclusion of social media variables seemed to capture protective and risky features of BSMM’s social lives that were not being captured in the self-reported data. Further research is needed to verify the acceptability and feasibility of incorporating social media data collection into established surveillance and prevention and treatment practice and identify ways to leverage insights from those efforts into near–real-time interventions.

## Appendix

Multimedia Appendix 1 Construction of topic dictionaries.

Multimedia Appendix 2 Multivariable logistic regression models stratified by HIV status.

## Abbreviations

BSMM: Black sexual minority men
OR: odds ratio
PrEP: pre-exposure prophylaxis
RDS: respondent-driven sampling
SMM: sexual minority men
STI: sexually transmitted infection
