# Supplementary material for: Social Media Communication and Network Correlates of HIV Infection and Transmission Risks Among Black Sexual Minority Men: Cross-sectional Digital Epidemiology Study
Source: JMIR Form Res. 2022 Oct 20;6(10):e37982. doi: 10.2196/37982 (PMC9634521; doi:10.2196/37982)
Supplement: Multimedia Appendix 1 [file formative_v6i10e37982_app1.docx]

**Multimedia Appendix 1**

***Constructing the Topic Dictionaries***

A mixed methods approach was used to build each HIV-related topic dictionary, which we depict visually in Figure 1. First, in a more deductive fashion, we turned to the literature to establish overarching topic categories centered around factors that are known correlates of HIV prevention and risk engagement among BSMM. Second, with topic categories established, we conducted semi-structured interviews with 15 BSMM to discuss how they and other members of their community talk about these topics on social media, focusing specifically on the terms, phrases, and symbols (e.g., emojis) they use to these ends. Third, we built on what we learned in the interviews with a more expansive internet search for slang, colloquialisms, and other informal terms and phrases that correspond to our topics of interest. With what we learned from the academic literature, interviews, and Internet sources, we built the first draft of each dictionary and used it to filter posts that included at least one term from the dictionary. After which, we examined frequently occurring bigrams that appear in the filtered posts from a given dictionary to see if any new words relevant to that topic emerged. The dictionaries were updated with any new words, and posts were filtered once more. The final step was to verify the overall accuracy of each dictionary through manual human checks on a random sample of the filtered posts. This enabled us to identify certain terms for which their inclusion in the dictionary introduced too much noise due to the term’s inherent double meaning, being an overly general term, or being reappropriated so as to mean something different than intended. Such terms were subsequently removed from each dictionary and posts were filtered again.

Figure 1. Schematic of the dictionary-building process

**
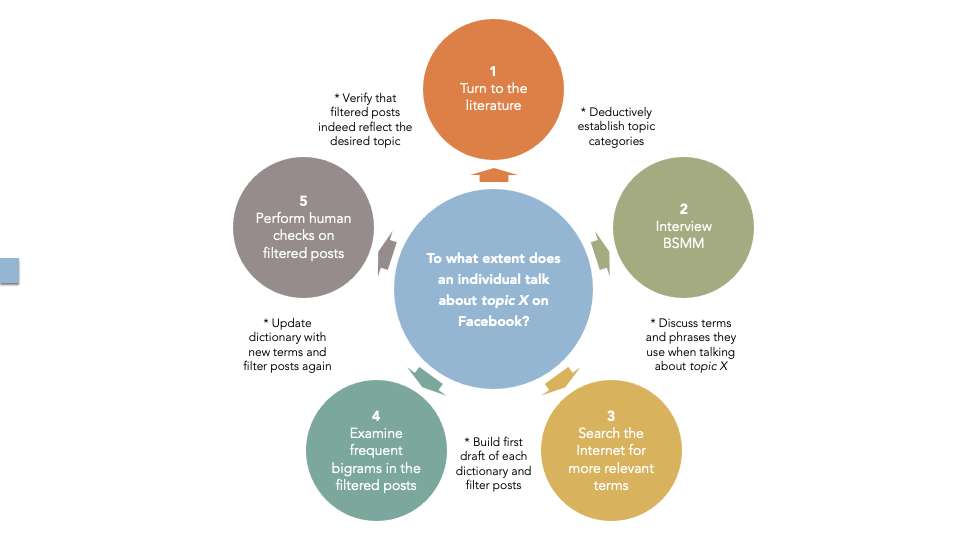
**

**References**

1. Bailey, M.M., *Performance as intravention: Ballroom culture and the politics of HIV/AIDS in Detroit.* Souls, 2009. **11**(3): p. 253-274.

2. Arnold, E. and M.M. Bailey, *Constructing Home and Family: How the Ballroom Community Supports African American GLBTQ Youth in the Face of HIV/AIDS.* J Gay Lesbian Soc Serv, 2009. **21**(2-3): p. 171-188.

3. Kubicek, K., et al., *“It’s like our own little world”: resilience as a factor in participating in the ballroom community subculture.* AIDS and Behavior, 2013. **17**(4): p. 1524-1539.
