# Supplementary material for: Social Media Communication and Network Correlates of HIV Infection and Transmission Risks Among Black Sexual Minority Men: Cross-sectional Digital Epidemiology Study
Source: JMIR Form Res. 2022 Oct 20;6(10):e37982. doi: 10.2196/37982 (PMC9634521; doi:10.2196/37982)
Supplement: Multimedia Appendix 2 [file formative_v6i10e37982_app2.docx]

**Multimedia Appendix 2**

**Table 1.** Multivariable logistic regression models to assess associations between Facebook communication and network features and engagement in biomedical prevention, stratified by HIV status

| Independent Variables | Full sample (n=310) | HIV negative (n=163) | HIV positive (n=147) |
| --- | --- | --- | --- |
|  | OR^a^ (95% CI) | OR (95% CI) | OR (95% CI) |
| **Facebook Communication Features** |  |  |  |
| Sexual health content (std)^b^ | 1.48 (1.00-2.20)^+^ | 1.53 (0.99-2.38)^+^ | 1.31 (0.67-2.55) |
| Substance use content (std) | 0.89 (0.58-1.36) | 0.70 (0.33-1.47) | 1.00 (0.46-2.17) |
| Sex behavior content (std) | 1.20 (0.82-1.77) | 1.11 (0.57-2.16) | 1.22 (0.75-1.97) |
| Ballroom culture content (std) | 0.06 (0.01-0.71)^*^ | 0.14 (0.01-2.14) | 0.01 (0.00-0.77)^*^ |
| Positive affect (std) | 0.85 (0.66-1.08) | 0.90 (0.60-1.35) | 0.82 (0.58-1.15) |
| **Facebook Network Features** |  |  |  |
| Eigenvector centrality (std) | 0.97 (0.69-1.36) | 1.34 (0.89-2.03) | 0.70 (0.45-1.09) |
| Brokerage (std) | 0.78 (0.44-1.37) | 0.85 (0.36-1.99) | 0.67 (0.32-1.42) |
| Local clustering coefficient (std) | 1.13 (0.76-1.66) | 0.89 (0.56-1.44) | 1.66 (0.77-3.60) |
| Facebook group count (std) | 0.85 (0.66-1.10) | 0.86 (0.48-1.56) | 0.89 (0.67-1.19) |
| **Controls** |  |  |  |
| HIV (positive) status | 3.20 (1.85-5.53)^***^ | -- | -- |
| Total number of posts (std) | 1.12 (0.68-1.84) | 1.00 (0.55-1.83) | 1.68 (0.76-3.70) |
| STI incidence | 2.65 (1.43-4.89)^**^ | 5.22 (1.98-13.75) ^***^ | 2.00 (0.86-4.63) |
| Condomless sex | 0.90 (0.52-1.57) | 1.44 (0.61-3.41) | 0.67 (0.30-1.48) |
| Sex drug use | 1.02 (0.59-1.76) | 0.97 (0.40-2.36) | 1.24 (0.56-2.77) |
| Depression (std) | 0.90 (0.69-1.17) | 0.97 (0.91-1.04) | 0.99 (0.93-1.06) |
| Intercept | 0.17 (0.09-0.35)^***^ | 0.15 (0.05-0.46) | 0.65 (0.22-1.91) |

^a^ OR: odds ratio

^b^ std: standard deviation unit change

^+^ p<.10

^*^ p<.05

^**^ p<.01

^***^ p<.001
